# Main roads to melanoma

**DOI:** 10.1186/1479-5876-7-86

**Published:** 2009-10-14

**Authors:** Giuseppe Palmieri, Mariaelena Capone, Maria Libera Ascierto, Giusy Gentilcore, David F Stroncek, Milena Casula, Maria Cristina Sini, Marco Palla, Nicola Mozzillo, Paolo A Ascierto

**Affiliations:** 1 Istituto di Chimica Biomolecolare, Consiglio Nazionale delle Ricerche (CNR), Sassari, Italy; 2 Istituto Nazionale Tumori "Fondazione Pascale", Napoli, Italy; 3 Cell Processing Section, Department of Transfusion Medicine Clinical Center, NIH, Bethesda, MD, USA

## Abstract

The characterization of the molecular mechanisms involved in development and progression of melanoma could be helpful to identify the molecular profiles underlying aggressiveness, clinical behavior, and response to therapy as well as to better classify the subsets of melanoma patients with different prognosis and/or clinical outcome. Actually, some aspects regarding the main molecular changes responsible for the onset as well as the progression of melanoma toward a more aggressive phenotype have been described. Genes and molecules which control either cell proliferation, apoptosis, or cell senescence have been implicated. Here we provided an overview of the main molecular changes underlying the pathogenesis of melanoma. All evidence clearly indicates the existence of a complex molecular machinery that provides checks and balances in normal melanocytes. Progression from normal melanocytes to malignant metastatic cells in melanoma patients is the result of a combination of down- or up-regulation of various effectors acting on different molecular pathways.

## Molecular complexity of melanoma pathogenesis

Melanocytic transformation is thought to occur by sequential accumulation of genetic and molecular alterations [1,2]. Although the pathogenetic mechanisms underlying melanoma development are still largely unknown, several genes and metabolic pathways have been shown to carry molecular alterations in melanoma.

A primary event in melanocytic transformation can be considered a cellular change that is clonally inherited and contributes to the eventual malignancy. This change occurs as a secondary result of some oncogenic activation through either genetic (gene mutation, deletion, amplification or translocation), or epigenetic (a heritable change other than in the DNA sequence, generally transcriptional modulation by DNA methylation and/or by chromatin alterations such as histone modification) events. The result of such a change would be the generation of a melanocytic clone with a growth advantage over surrounding cells. Several pathways have been found to be involved in primary clonal alteration, including those inducing the cell proliferation (*proliferative pathways*) or overcoming the cell senescence (*senescence pathway*). Conversely, reduced apoptosis is highly selective or required for the development of advanced melanoma (*apoptotic pathways*).

### Proliferative pathways

The MAPK-ERK pathway (including the cascade of NRAS, BRAF, MEK1/2, and ERK1/2 proteins), a major signaling cascade involved in the control of cell growth, proliferation and migration, has been reported to play a major role in both the development and progression of melanoma (the increased activity of ERK1/2 proteins, which have been found to be constitutively activated in melanomas mostly as a consequence of mutations in upstream components of the pathway) and seems to be implicated in rapid melanoma cell growth, enhanced cell survival and resistance to apoptosis [3,4].

A less common primary pathway which stimulates cell proliferation, without MAPK activation, seems to be the reduction of RB (retinoblastoma protein family) activity by *CyclinD1 *or *CDK4 *amplification or RB mutation (impaired RB activity through increased CDK4/cyclin D1 could substitute for the MAPK activation and initiate clonal expansion) [4,5].

### Senescence pathways

Cell senescence is an arrest of proliferation at the somatic level, which is induced by telomere shortening, oncogenic activation, and/or cellular stress due to intense proliferative signals [6,7]. In recent years, a common mechanism for the induction of cell senescence has been described: a progressive-reduction in the length of telomeres (often, in conjunction with overactivity of specific oncogenes - such as *MYC *and *ATM*) seems to exert DNA damage signaling with activation of the p16^CDKN2A ^pathway [8,9]. Nevertheless, cancers including melanomas cannot grow indefinitely without a mechanism to extend telomeres. The expression and activity of telomerase is indeed up-regulated in melanoma progression [10]. This evidence strongly suggests that both telomere length and p16^CDKN2A ^act in a common pathway leading to growth-arrest of nevi. In particular, the p16^CDKN2A ^protein acts as an inhibitor of melanocytic proliferation by binding the CDK4/6 kinases and blocking phosphorylation of the RB protein, which leads to cell cycle arrest [11]. Dysfunction of the proteins involved in the p16^CDKN2A ^pathway have been demonstrated to promote uncontrolled cell growth, which may increase the aggressiveness of transformed melanocytic cells [12].

### Apoptotic pathways

The p14^CDKN2A ^protein exerts a tumor suppressor effect by inhibiting the oncogenic actions of the downstream MDM2 protein, whose direct interaction with p53 blocks any p53-mediated activity and targets the p53 protein for rapid degradation [13]. Impairment of the p14^CDKN2A^-MDM2-p53 cascade, whose final effectors are the Bax/Bcl-2 proteins, has been implicated in defective apoptotic responses to genotoxic damage and, thus, to anticancer agents (in most cases, melanoma cells present concurrent high expression levels of Bax/Bcl-2 proteins, which may contribute to further increasing their aggressiveness and refractoriness to therapy) [14,15].

## The main genes and related pathways in melanoma

### *BRAF*

Exposure to ultraviolet light is an important causative factor in melanoma, although the relationship between risk and exposure is complex. Considerable roles for intermittent sun exposure and sunburn history in the development of melanoma have been identified in epidemiologic studies [16].

The pathogenic effects of sun exposure could involve the genotoxic, mitogenic, or immunosuppressive responses to the damage induced in the skin by UVB and UVA [17,18]. UVB represents only a small portion of the solar radiation reaching the earth's surface (<5%) but it can directly damage DNA through mutagenesis at dipyrimidine sites, inducing apoptosis in keratinocytes. UVA indirectly damages DNA primarily through the generation of reactive oxygen species and formation of 8-oxo-7,8-dihydro-2'-deoxyguanosine. These reactive oxygen species subsequently damage DNA especially by the formation of G>T transversion mutations [19].

It is controversial as to whether the UVB or the UVA component of solar radiation is more important in melanoma development [20,21]. One of the major reasons for this uncertainty is that sunlight is a complex and changing mix of different UV wavelengths, so it is very difficult to accurately delineate the precise lifetime exposures of individuals and entire populations to UVA and UVB from available surrogates, such as latitude at diagnosis or exposure questionnaires [19]. A significant body of epidemiological evidence suggests that both UVA and UVB are involved in melanoma causation [20-24].

The clinical heterogeneity of melanoma can probably be explained by the existence of genetically distinct types of melanoma with different susceptibility to ultraviolet light [5]. Cutaneous melanomas, indeed, have four distinct subtypes:

- *Superficial Spreading Melanoma *(SSM), on intermittently exposed skin (i.e., upper back);

- *Lentigo Maligna Melanoma *(LMM), on chronically exposed skin;

- *Acral Lentiginous Melanoma *(ALM), on the hairless skin of the palms and soles;

- *Nodular Melanoma *(NM), with tumorigenic vertical growth, not associated with macular component [25].

From a molecular point of view, the signaling cascades involving the *melanocortin-1-receptor *(MC1R) and RAS-BRAF genes have been demonstrated to represent a possible target of UV-induced damage.

The *MC1R *gene encodes the melanocyte-stimulating hormone receptor (MSHR), a member of the G-protein-coupled receptor superfamily which normally signals the downstream BRAF pathway by regulating intracellular levels of cAMP [26,27]. The *MC1R *gene is remarkably polymorphic in Caucasian populations, representing one of the major genetic factors which determines skin pigmentation. Its sequence variants can result in partial (r) or complete (R) loss of the receptor's signalling ability [28,29]. The *MC1R *variants have been suggested to be associated with red hair, fair skin, and increased risk of both melanoma and non-melanoma skin cancers [29,30].

RAS and BRAF are two important molecules belonging to the *mitogen-activated protein kinase *(MAPK) signal transduction pathway, which regulates cell growth, survival, and invasion. MAPK signaling is initiated at the cell membrane, either by receptor tyrosine kinases (RTKs) binding ligand or integrin adhesion to extracellular matrix, which transmits activation signals via the RAS-GTPase on the cell membrane inner surface. Active, GTP-bound RAS can bind effector proteins such as RAF serine-threonine kinase or phosphatidylinositol 3-Kinase (PI3K) [31,32].

In mammals, three highly conserved *RAF *genes have been described: *ARAF*, *BRAF*, and *CRAF *(*Raf-1*). Although each isoform possesses a distinct expression profile, all RAF gene products are capable of activating the MAPK pathway [33,34]. *CRAF *and *ARAF *mutations are rare or never found in human cancers [35-37]. This is probably related to the fact that oncogenic activation of *ARAF *and *CRAF *require the coexistence of two mutations [34,36]. The *BRAF *gene, which can conversely be activated by single amino acid substitutions, is much more frequently mutated in human cancer (approximately 7% of all types). Activating mutations of *BRAF *have been found in colorectal, ovarian [3], thyroid [38], and lung cancers [39] as well as in cholangiocarcinoma [40], but the highest rate of *BRAF *mutations (overall, about half of cases) have been observed in melanoma [41].

The most common mutation in *BRAF *gene (nearly, 90% of cases) is a substitution of valine with glutamic acid at position 600 (V600E) [3]. This mutation, which is present in exon 15 within the kinase domain, activates *BRAF *and induces constitutive MEK-ERK signaling in cells [3,42]. The activation of *BRAF *leads to the downstream expression induction of the *microphthalmia-associated transcription factor *(*MITF*) gene, which has been demonstrated to act as the master regulator of melanocytes. Activated *BRAF *also participates in the control of cell cycle progression (see below) [43].

Activating *BRAF *mutations have been detected in melanoma patients only at the somatic level [44] and in common cutaneous nevi [45]. Among primary cutaneous melanomas, the highest prevalence of *BRAF *oncogenic mutations has been reported in late stage tumors (mostly, vertical growth phase lesions) [46,47]. Therefore, the role of *BRAF *activation in pathogenesis of melanoma remains controversial.

The presence of *BRAF *mutations in nevi strongly suggests that BRAF activation is necessary but not sufficient for the development of melanoma (also known as melanomagenesis). To directly test the role of activated *BRAF *in melanocytic proliferation and transformation, a transgenic zebrafish expressing BRAF-V600E presented a dramatic development of patches of ectopic melanocytes (termed as fish-nevi) [48]. Remarkably, activated *BRAF *in p53-deficient zebrafish induced the formation of melanocytic lesions that rapidly developed into invasive melanomas, which resembled human melanomas in terms of histological and biological behaviors[48]. These data provide direct evidence that the p53 and BRAF pathways functionally interact to induce melanomagenesis. *BRAF *also cooperates with *CDKN2A*, which maps at the CDKN locus and encodes two proteins: the cyclin-dependent kinase inhibitor p16^CDKN2A^, which is a component of the CyclinD1-RB pathway, and the tumor suppressor p14^CDKN2A^, which has been functionally linked to the MDM2-p53 pathway (see below). Activating *BRAF *mutations have been reported to constitutively induce up-regulation of *p16*^*CDKN*2*A *^and cell cycle arrest (this phenomenon appears to be a protective response to an inappropriate mitogenic signal) [4,49]. In particular, mutant BRAF protein induces cell senescence by increasing the expression levels of the p16^CDKN2A ^protein, which, in turn, may limit the hyperplastic growth caused by *BRAF *mutations [49]. Recently, it has been demonstrated that other factors, such as those regulated by the IGFBP7 protein, may participate in inducing the arrest of the cell cycle and cell senescence caused by the *BRAF *activation [50-52]. As for p53 deficiency, a genetic or epigenetic inactivation of *p16*^*CDKN*2*A *^gene and/or alterations of additional cell-cycle factors may therefore contribute to the *BRAF*-driven melanocytic proliferation.

The observation that early stage melanomas exhibit a lower prevalence of *BRAF *mutations than that found in late stage lesions [46,47] argues against the hypothesis that *BRAF *activation participates in the initiation of melanoma but seems to strongly suggest that such an alteration could be involved in disease progression. Moreover, similar rates of *BRAF *mutations have been reported in various histological types of nevi (including congenital, intradermal, compound, and atypical ones) [45], suggesting that the activation of *BRAF *does not likely contribute to possible differences in the propensity to progression to melanoma among these nevi subsets. Taken together, all of this evidence, strongly suggests that activating *BRAF *mutations induce cell proliferation and cell survival, which represent two biological events occurring in both melanocytic expansion of nevi and malignant progression from superficial to invasive disease.

Finally, *BRAF *mutations occur at high frequency in melanomas that are strongly linked to intermittent sun exposure (non Chronic Sun-induced Damage, non-CSD), though sun exposure has not been shown to directly induce the T1796→A transition underlying the V600E change at exon 15. In fact, this transition does not affect a dipyrimidine site and cannot be considered to be the result of a UVB-induced replication error. Further work is needed to better understand the interaction of UV exposure and *BRAF *mutations. Recently, *MC1R *variants have been strongly associated with *BRAF *mutations in non-CSD melanoma, which has lead to the hypothesis that *BRAF *activation may be somehow indirectly induced by UV radiation [53]. In this regard, mutations in the upstream gene *NRAS *which occur in about 15% of cutaneous melanomas (*NRAS *and *BRAF *mutations are mutually exclusive in the same tumor, suggesting functional redundancy [5,54]), have been rarely found in melanoma lesions arising in sun-exposed sites; they do not correlate with the degree of sun exposure, histologic subtype, or anatomical site [55,56].

Other distinct subgroups of melanoma have been shown to harbor oncogenic mutations in the receptor tyrosine kinase *KIT*. While *BRAF *mutations are the most common oncogenic mutation in cutaneous melanoma, mucosal melanomas and acral lentiginous melanomas often have wild type *BRAF*, but may carry mutations in *KIT *gene (though, the role of such alterations in melanomagenesis are yet to be clearly defined). In most cases, *KIT *mutations are accompanied by an increase in gene copy number and genomic amplification [57,58].

### *CDKN2A *and *CDK4*

The Cyclin-Dependent Kinase Inhibitor 2A (*CDKN2A*, also called Multi Tumor-Suppressor MTS1) [59] is the major gene involved in melanoma pathogenesis and predisposition. It is located on chromosome 9p21 and encodes two proteins, p16^CDKN2A ^(including exons 1α, 2 and 3) and p14^CDKN2A ^(a product of an alternative splicing that includes exons 1β and 2) [60,61], which are known to function as tumour suppressors. The p16^CDKN2A ^and p14^CDKN2A ^are simultaneously altered in multiple tumors since most of their pathogenetic mutations occur in exon 2, which is encoded in both gene products. The inactivation of *CDKN2A *is mostly due to deletion, mutation or promoter silencing (through hypermethylation).

The p16^CDKN2A ^protein inhibits the activity of the cyclin D1-cyclin-dependent kinase 4 (CDK4) complex, whose function is to drive cell cycle progression by phosphorylating the retinoblastoma (RB) protein. Thus, p16^CDKN2A ^induces cell cycle arrest at G1 phase, blocking the RB protein phosphorylation. On this regard, RB phosphorylation causes the release of the E2F transcription factor, which binds the promoters of target genes, stimulating the synthesis of proteins necessary for cell division. Normally the RB protein, through the binding of E2F, prevents the cell division. When the RB protein is absent or inactivated by phosphorilation, E2F is available to bind DNA and promote the cell cycle progression [62].

p14^CDKN2A ^stabilizes p53, interacting with the Murine Double Minute (MDM2) protein, whose principal function is to promote the ubiquitin-mediated degradation of the p53 tumor suppressor gene product [63-66]. The shuttling of p53 by MDM2 from nucleus to cytoplasm is required for p53 to be subject to proteosome-mediated degradation. The p53 protein has been named "guardian of the genome", because it arrests cell division at G1 phase to allow DNA repair or to induce apoptosis of potentially transformed cells. In normal conditions, the expression levels of p53 in cells are low. In response to DNA damage, p53 accumulates and prevents cell division. Therefore, inactivation of the *TP53 *gene results in an accumulation of genetic damage in cells which promotes tumor formation [67]. In melanoma, such an inactivation is mostly due to a functional gene silencing since the frequency of *TP53 *mutations is low [68]. Different signals regulate p53 levels by controlling its binding with MDM2. Several kinases play this role, catalyzing stress-induced phosphorylation of serine in the trans-activation domain of p53. Moreover, several proteins, including E2F, stabilize p53 through the p14^CDKN2A^-mediated pathway. The interaction of protein p300 with MDM2 promotes p53 degradation.

Data obtained from genetic and molecular studies over the past few years have indicated that the CDKN2A locus as the principal and rate-limiting target of UV radiation in melanoma formation [69]. *CDKN2A *has been designated as a high penetrance melanoma susceptibility gene [70]; however, the penetrance of its mutations is influenced by UV exposure [71] and varies according to the incidence rates of melanoma in different populations (indeed, the same factors that affects population incidence of melanoma may also mediate *CDKN2A *mutation penetrance). The overall prevalence of melanoma patients who carry a *CDKN2A *mutation is between 0.2% and 2%. The penetrance of *CDKN2A *mutations is also greatly influenced by geographic location, with reported rates of 13% in Europe, 50% in the US, and 32% in Australia by 50 years of age; and 58% in Europe, 76% in the US, and 91% in Australia by age 80 [72].

*CDKN2A *mutations are more frequent in patients with a strong familial history of melanoma (three or more affected family members; 35.5%) [73] compared with patients without any history (8.2%). Moreover, the frequency of *CDKN2A *mutations is also higher in patients with synchronous or asynchronous multiple melanomas (more than two diagnosed lesions, 39.1%; only two melanomas, 10%) [72]. Although families identified with *CDKN2A *mutations display an average disease penetrance of 30% by 50 years of age and 67% by age 80, studies have shown that melanoma risk is greatly influenced by the year an individual is born, levels of sun exposure, and other modifier genes.

Correlations between the *CDKN2A *mutation status and melanoma risk factors in North American melanoma-prone families have shown that in addition to the increased risk associated with *CDKN2A *mutations, the total number of nevi and the presence of dysplastic nevi were associated with a higher risk of melanoma, Sun exposure and a history of sunburn is associated with melanoma risk in melanoma-prone families. In other words, the melanoma risk associated with sunburn was higher in individuals in genetically susceptible families than in non-susceptible individuals. This finding suggests that there are common mechanisms and/or interactions between the *CDKN2A *pathway and the UV-sensitivity [72]. Many high-risk families exhibit atypical nevus/mole syndrome (AMS) characterized by atypical nevi, increased banal nevi and atypical nevus distribution on ears, scalp, buttocks, dorsal feet and iris. In a study of *CDKN2A *mutation carriers, a similar distribution was present on buttocks and feet, and in a p16^CDKN2A ^family with a temperature-sensitive mutation, nevi were found to be distributed in warmer regions of the body (head, neck and trunk). This supports the hypothesis that p16^CDKN2A ^mutations play a role in nevus senescence.

The second melanoma susceptibility gene is the Cyclin-Dependent Kinase 4, which is located at 12q13.6, and which encodes a protein interacting with the *p16*^*CDKN*2*A *^gene product. CDK4 is a rare high-penetrance melanoma predisposition gene. Indeed, only three melanoma families worldwide are carriers of mutations in *CDK4 *(Arg24Cys and Arg24His). From a functional point of view, the Arg24Cys mutation, located in the p16^CDKN2*A*^-binding domain of CDK4, make the p16^CDKN2A ^protein unable to inhibit the D1-cyclin-CDK4 complex, resulting in a sort of oncogenic activation of *CDK4*.

### *PTEN *and *AKT*

The *PTEN *gene (phosphatase and tensin homolog deleted on chromosome 10) is located at the chromosome 10q23.3 [74] and is mutated in a large fraction of human melanomas. The protein encoded by this gene acts as an important tumor suppressor by regulating cellular division, cell migration and spreading [75], and apoptosis [76-78] thus preventing cells from growing and dividing too rapidly or in an uncontrolled way. The PTEN protein has at least two biochemical functions: lipid phosphatase and protein phosphatase. The lipid phosphatase activity of PTEN seems to have a role in tumorigenesis by inducing a decrease in the function of the downstream AKT protein (also knows as protein kinase B or PKB). In particular, the most important effectors of PTEN lipid phosphatase activity are phosphatidylinositol-3,4,5-trisphosphate (PIP3) and phosphatidylinositol 3,4-bisphosphate (PIP2) that are produced during intracellular signaling by the activation of lipid kinase phoshoinosite 3-kinase (PI3K). PI3K activation results in an increase of PIP3 and a consequent conformational change activating AKT [79]. This latter protein is a serine/threonine kinase and belongs to the AKT protein kinase family: AKT1, AKT2, and AKT3. Although all AKT isoforms may be expressed in a different cell type, they share a high degree of structural similarity [80-83]. Under physiologic circumstances, the PI3K/PTEN/AKT pathway is triggered by paracrine/autocrine factors (e.g., insulin-like growth factor-I) [84].

Moreover, recent studies have also revealed a role for AKT in the activation of NF-kB which is considered to be an important pleiotropic transcription factor involved in the control of cell proliferation and apotosis in melanoma. Upon activation, NF-kB can regulate the transcription of a wide variety of genes, including those involved in cell proliferation. It has been reported that PTEN expression is lost in melanoma cell lines with high AKT expression, suggesting that the activation of AKT induced by PTEN inactivation or growth factor signaling activation could represent an important common pathway in the progression of melanoma (probably, by enhancing cell survival through up-regulation of NF-kB and escape from apoptosis) [85].

AKT activation stimulates cell cycle progression, survival, metabolism and migration through phosphorylation of many physiological substrates [86-90]. Based on its role as a key regulator of cell survival, AKT is emerging as a central player in tumorigenesis. It has been proposed that a common mechanism of activation of AKT is DNA copy gain involving the AKT3 locus, which is found in 40-60% of melanomas. AKT3 expression strongly correlates with melanoma progression, and depletion of AKT3 induces apoptosis in melanoma cells and reduces the growth of xenografts [91-93]. Mutations in the gene encoding the catalytic subunit of PI3K (*PIK3CA*) occur at high frequencies in some human cancers [94], leading to constitutive AKT activation [95] but occur at very low rates (5%) in melanoma [96,97]. Activated AKT seems to promote cell proliferation, possibly through the down-regulation of the cyclin-dependent kinase inhibitor p27 as well as the up-regulation and stabilization of cyclin D1 [98]. The activation of AKT also results in the suppression of apoptosis induced by a number of stimuli including growth factor withdrawal, detachment of extra-cellular matrix, UV irradiation, cell cycle discordance, and activation of FAS signaling [88,99-101]. The mechanisms associated with the ability of AKT to suppress apoptosis [89,99-101] include the phosphorylation and inactivation of many pro-apoptotic proteins, such as BAD (Bcl-2 antagonist of cell death, a Bcl2 family member [101]), caspase-9 [102], MDM2 (that lead to increased p53 degradation [103-105]), and the forkhead family of transcription factors [106], as well as the activation of NF-kB [107]. It has been proposed that UV irradiation induces apoptosis in human keratinocytes *in vitro *and *in vivo*, and also activates survival pathways including PIP3 kinase and its substrate AKT, in order to limit the extent of cell death [108]. A direct correlation between radiation resistance and levels of PI3K activity has been indeed described. Although activating mutations of *AKT *are nearly absent in melanoma (a rare mutation in *AKT1 *and *AKT3 *genes has been recently reported in a limited number of human melanomas and melanoma cell lines [109-111], the silencing of AKT function by targeting PI3K inhibits cell proliferation and reduces sensitivity of melanoma cells to UV radiation [112].

The lipid phosphatase activity of PTEN protein is able to degrade the products of PI3K [113], suggesting that PTEN functions may directly antagonize the activity of P13K/AKT pathway [114,115]. As predicted by this model, genetic inactivation of PTEN in human cancer cells leads to constitutive activation of this AKT pathway and mediates tumorigenesis. Numerous mutations and/or deletions in the PTEN gene have been found in tumours including lymphoma; thyroid, breast, and prostate carcinomas;, and melanoma [116-118]. PTEN somatic mutations are found in 40-60% of melanoma cell lines and 10-20% of primary melanomas [119]. The majority of such mutations occurs in the phosphatase domain [117,118]. The contrast between the detection of a low mutation frequency and a higher level of gene silencing in primary melanomas has led to speculate that *PTEN *inactivation may predominantly occur through epigenetic mechanisms [120]. Several distinct methylation sites have been found within the PTEN promoter and hypermethylation at these sites has been demonstrated to reduce the PTEN expression in melanoma. PTEN is involved in the inhibition of focal adhesion formation, cell spreading and migration as well as in the inhibition of growth factor-stimulated MAPK signaling (alterations in the BRAF-MAPK pathway are frequently associated with PTEN-AKT impairments [8,121]). Therefore, the combined effects of the loss of the PTEN function may result in aberrant cell growth, escape from apoptosis, and abnormal cell spreading and migration. In melanoma, PTEN inactivation has been mostly observed as a late event, although a dose-dependent down-regulation of PTEN expression has been implicated in early stages of tumorigenesis. In addition, loss of PTEN protein and oncogenic activation of NRAS seem to be mutually exclusive and both alterations may cooperate with the loss of CDKN2A expression in contributing to melanoma tumorigenesis [122].

### *MITF*

Increased interest has been focused on the activity of the microphthalmia-associated transcriptor factor (MITF), which is considered to be the "master regulator of melanocytes" since it seems to be crucial for melanoblast survival and melanocyte lineage commitment.

MITF maps on chromosomre 3p14.1-p12.3 and encodes for a basic helix-loop-helix (hHLH)-leucine zipper protein that plays a role in the development of various cell types, including neural crest-derived melanocytes and optic cup-derived retinal pigment epithelial cells [123]. MITF was first identified in the mouse as a locus whose mutation results in the absence of pigment cells causing white coat color and deafness due to melanocyte deficiency in the inner ear [124]. In humans, mutation of MITF results in Waardenburg Syndrome IIa, a condition characterized by white forelock and deafness [125]. A role for MITF in pigment gene regulation has been suggested [126-129], based on the existence of highly conserved MITF consensus DNA binding elements in the promoters of major pigment enzyme genes: tyrosinase, Tyrp1, Dct, and pmel17 (all involved in the functional differentiation of melanocytes) [130]. Transfection of MITF into cell lines has indicated a regulatory activity of the transfected MITF construct on the regulation of the pigmentation pathways [131]. Increasing evidence also suggests a role for MITF in the commitment, proliferation, and survival of melanocytes before and/or during neural crest cell migration [132]. These studies suggest that MITF, in addition to its involvement into the differentiation pathways such as pigmentation, may play an important role in the proliferation and/or survival of developing melanocytes, contributing to melanocyte differentiation by triggering cell cycle exit.

The differentiation functions of MITF are displayed when the expression levels of this protein are high. Indeed, high MITF levels have been demonstrated to exert an anti-proliferative activity in melanoma cells [133]. In this regard, low levels of MITF protein were found in invasive melanoma cells [134] and have been associated with poor prognosis and clinical disease progression [131,135,136]. In a multivariate analysis, the expression of MITF in intermediate-thickness cutaneous melanoma was inversely correlated with overall survival [135]. The authors speculated that MITF might be a new prognostic marker in intermediate-thickness malignant melanoma. The retention of MITF expression in the vast majority of human primary melanomas, including non-pigmented tumors, is consistent with this hypothesis and has also led to the widespread use of MITF as a diagnostic tool in this malignancy [135,137-139]. The MITF gene has been found to be amplified in 15% to 20% of metastatic melanomas [140-142]. In melanomas, MITF targets a number of genes with antagonistic behaviors, including genes such as CDK2 and Bcl-2, which promote cell cycle progression and survival, as well as p21^CIP1 ^and p16^INK4A^, which halt the cell cycle [43,143-145]. Furthermore, MITF resides downstream of two key anti-apoptotic pathways, the ERK and the PI3-kinase pathways, suggesting that MITF could integrate extracellular pro-survival signals [146]. Overall, the question of whether MITF may exert a pro-survival effect or growth inhibition in melanocytes and melanoma is still open and not yet fully understood. One could speculate that the cellular context and microenvironment may represent important influencing factors.

The expression and function of MITF can be regulated by a variety of cooperating transcription factors, such as Pax3, CREB, Sox10, Lef1, and Brn-2 [146,147] as well as by members of the MAPK and cAMP pathways [148-150]. In melanoma cells, activated BRAF suppresses MITF protein levels through ERK-mediated phosphorylation and degradation [133]. Furthermore, the *MITF *gene is amplified in 10-15% of melanomas carrying a mutated BRAF [141], supporting the view that continued expression of MITF is essential in melanoma cells. MITF was recently shown to also act downstream of the canonical WNT pathway, which includes cysteine-rich glycoproteins that play a critical role in development and oncogenesis [151]. In particular, the WNT gene family has been demonstrated to be involved into the development of the neural crest during melanocyte differentiation from pluripotent cells among several species (from zebrafish to mammalians) [151-154]. Moreover, several WNT proteins have been shown to be overexpressed in various human cancers; among them, the up-regulation of the WNT2 seems to participate in inhibiting normal apoptotic machinery in melanoma cells [155] (recently, it has been suggested that the WNT2 protein expression levels can be also useful in the differential diagnosis of nevus versus melanoma [156]). A key downstream effector of this pathway is β-catenin. In the absence of WNT-signals, β-catenin is targeted for degradation through phosphorylation controlled by a complex consisting of glycogen synthase kinase-3-beta (GSK3β), axin, and adenomatous polyposis coli (APC) proteins. The WNT signals lead to the inactivation of GSK3β, thus stabilizing the intracellular levels of β-catenin and subsequently increasing transcription of downstream target genes. Mutations in multiple components of the WNT pathway have been identified in many human cancers, all of the mutations induce nuclear accumulation of β-catenin [151,157]. In human melanoma, stabilizing mutations of β-catenin have been found in a significant fraction of established cell lines. Almost one third of these cell lines display aberrant nuclear accumulation of β-catenin, although few mutations have been classified as pathogeneic variants [157,158]. These observations are consistent with the hypothesis that this pathway contributes to behavior of melanoma cells and might be inappropriately deregulated for the development of the disease.

**Figure 1:**
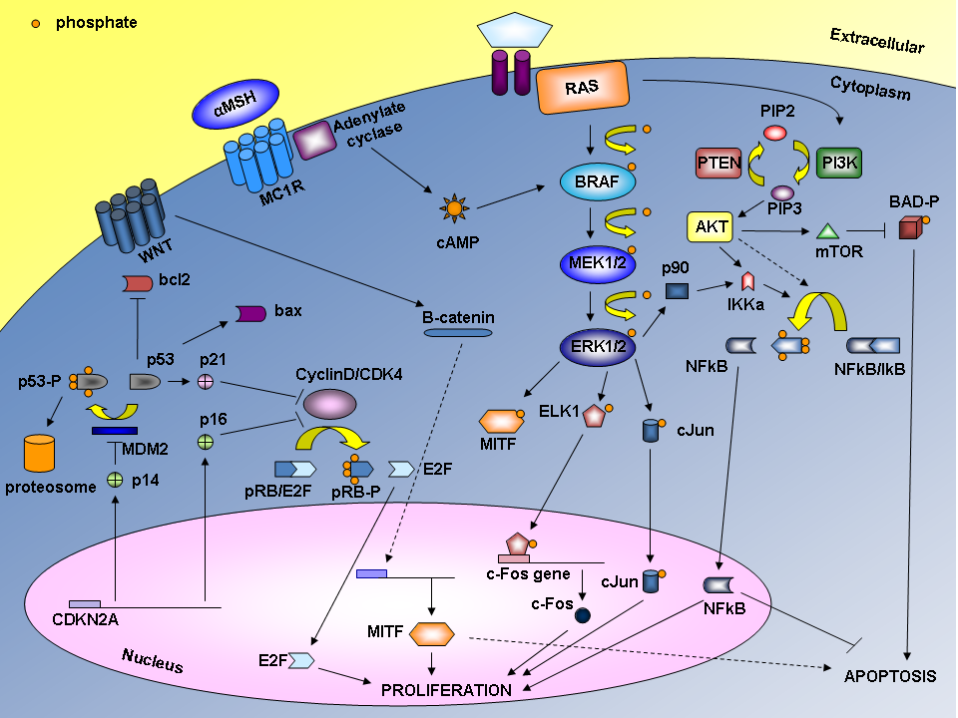
**Major pathways involved in melanoma**. Pathway associated with N-RAS, BRAF, and mitogen-activated protein kinase (MAPK) as well as with CDKN2A and MITF are schematically represented. Arrows, activating signals; interrupted lines, inhibiting signals. BAD, BCL-2 antagonist of cell death; cAMP, cyclic AMP; CDK4, Cyclin-dependent kinase 4; CDKN2A, Cyclin-dependent kinase inhibitor of kinase 2A; ERK1/2, Extracellular-related kinase 1 or 2; IkB, inhibitor of kB protein; IKK, inhibitor-of-kB-protein kinase; MC1R, melanocortin-1-receptor; MITF, Microphthalmia-Associated Transcription Factor; MEK1/2, Mitogen-activated protein kinase-extracellular related kinase 1/2; PI3K, Phosphatidylinositol 3 kinase; PIP2, Phosphatidylinositol bisphosphate; PIP3, Phosphatidylinositol trisphosphate; PTEN, Phosphatase and tensin homologue.

In Figure 1, the main effectors of all the above-mentioned pathways with their functional relationships are schematically reported.

## Novel signaling pathways in melanoma

### Notch1

**Figure 2:**
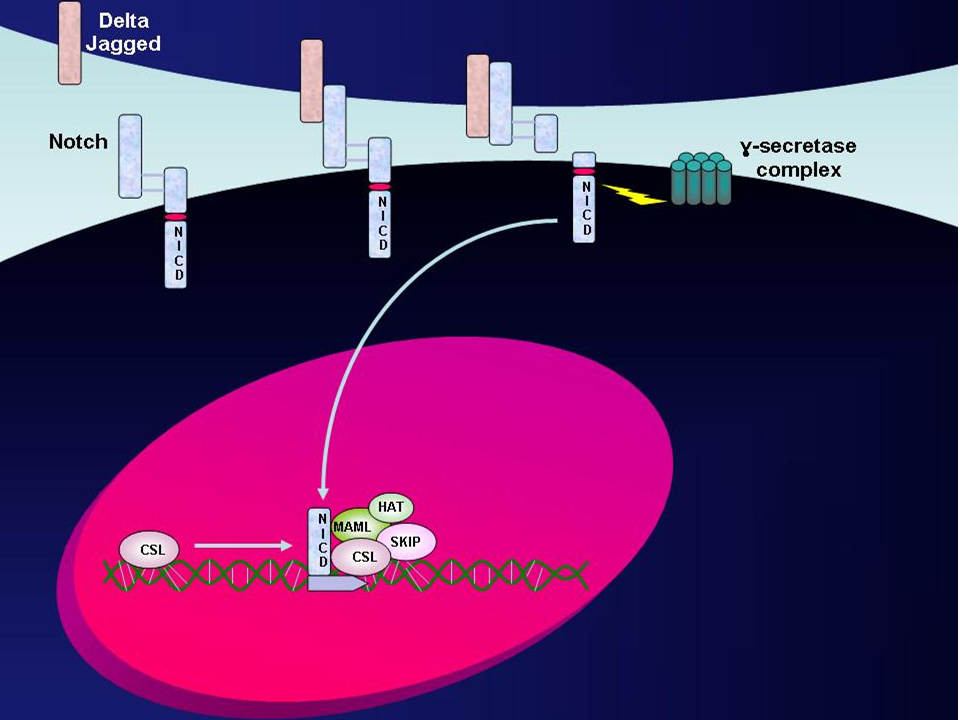
**Notch1 pathway**. The diagram shows the mechanism of activation of the Notch receptor by a cell-cell interaction through specific trasmembrane ligands, followed by the translation of the intracellular domain of the Notch-1 receptor (NICD) and formation of a transcription-activating multimeric complex. CSL, citrate synthase like; HAT, histone acetyltransferase; MAML, mastermind-like protein; SKIP, Skeletal muscle and kidney-enriched inositol phosphatase.

Notch proteins are a family of a single-pass type I transmembrane receptor of 300 kDa that was first identified in Drosophila *melanogaster *(at this level, a mutated protein causes 'notches' in the fly wing [159]). In vertebrates, there are four *Notch *genes encoding four different receptors (Notch1-4) that differ by the number of epidermal growth factor-like (EGF-like) repeats in the extracellular domain, as well as by the length of the intracellular domain [160-162]. These receptors are activated by specific transmembrane ligands which are expressed on an adjacent cell and activate Notch signaling through a direct cell-cell interaction (Figure 2). When a cell expressing a Notch receptor is stimulated by the adjacent cell via a Notch ligand on the cell surface, the extracellular subunit is trans-endocytosed in the ligand-expressing cell. The remaining receptor transmembrane subunit undergoes two consecutive enzymatic cleavages. The first activating cleavage is mediated by a metalloprotease-dependent TNF-α Converting Enzyme (TACE) [163,164]. This step is rapidly followed by a second cleavage in the transmembrane domain to generate an intracellular truncated version of the receptor designated as N^ICD^. Thus, the rate of cleavage of Notch-1 is finely modulated by multiple post-translational modifications and cellular compartmentalization events. The intracellular domain of the Notch-1 receptor (N^ICD^) can be then moved to the nucleus, where it forms a multimeric complex with a highly conserved transcription factor (CBF1, a repressor in the absence of Notch-1), and other transcriptional co-activators that influence the intensity and duration of Notch signals (Figure 2) [165,166]. The final result is the activation of transcription at the level of promoters containing CBF-1-responsive elements, thus stimulating or repressing the expression of various target genes [167].

The Notch signaling pathway plays a pivotal role in tissue homeostasis and regulation of cell fate, such as self-renewal of adult stem cells, as well as in the differentiation of precursors along a specific cell lineage [168-170]. Increasing evidence suggests its involvement in tumorigenesis, since deregulated Notch signaling is frequently observed in a variety of human cancers, such as T-cell acute lymphoblastic leukemias [171], small cell lung cancer [172], neuroblastoma [173,174], cervical [175,176] and prostate carcinomas [177]. *Notch *can act as either an oncogene or a tumor suppressor depending on both cellular and tissue contexts. Many studies suggest a role for Notch1 in keratinocytes as a tumor suppressor [178]. In such cells, Notch signaling induces cell growth arrest and differentiation (deletion of Notch1 in murine epidermis causes epidermal hyperplasia and skin carcinoma) [179,180]. The anti-tumor effect of Notch1 in murine skin appears to be mediated by p21^Waf1/Cip^induction and repression of WNT signaling [151,178].

Unlike keratinocyte-derived squamous cell and basal cell carcinomas, melanomas have a significantly higher Notch activity in comparison with normal melanocytes [181,182]. Investigation of the expression of Notch receptors and their ligands in benign and malignant cutaneous melanocytic lesions indicate that Notch1 and Notch2, as well as their ligands are significantly upregulated in atypical nevi and melanomas, compared to common melanocytic nevi [181,182]. Furthermore, a constitutively-induced gene activation in human melanocytes strongly suggests that *Notch1 *acts as a transforming oncogene in such a cell lineage [183]. The versatile effects of Notch1 signaling on cell differentiation, proliferation, survival, and tumorigenesis may easily explain why *Notch1 *plays different roles in various types of skin cancers. Such different activities of *Notch1 *in skin cancer are probably determined by its interaction with the downstream β-catenin target. In murine skin carcinoma, β-catenin is functional activated by Notch1 signaling and mediates tumor-suppressive effects [178,184]. In melanoma, β-catenin mediates oncogenic activity by also cross-talking with the WNT pathway or by regulating N-cadherin, with different effects on tumorigenesis depending on *Notch1 *activation [185].

Recent evidence suggest that Notch1 enhances vertical growth phase by the activation of the MAPK and AKT pathways; inhibition of either the MAPK or PI3K-AKT pathway reverses the tumor cell growth induced by Notch1 signaling. Future studies aimed at identifying new targets of Notch1 signaling will allow the assessment of the mechanisms underlying the crosstalk between Notch1, MAPK, and PI3K-AKT pathways. Finally, Notch signaling can enhance the cell survival by interacting with transcriptional factor NF-kB (N^IC ^seems to directly interact with NF-kB, leading to retention of NF-kB in the nucleus of T cells) [186]. Nevertheless, it has been shown that N^IC ^can directly regulate IFN-γ expression through the formation of complexes between NF-kB and the IFN-γ promoter. Although there is a lack of consensus about crosstalk between Notch1 and NF-kB, existing data suggest that two mechanisms of NF-kB activation may occur: an early Notch-independent phase and a late Notch-dependent activation of NF-kB [187]. Finally, RAS-mediated transformation requires the presence of intact Notch signaling; impairment of such Notch1 receptor signaling may significantly reduce the ability of RAS to transform cells [188,189].

In conclusion, although the precise details of the mechanisms by which Notch1 signaling can contribute to melanoma development remain to be defined, *Notch1 *could be clearly considered as a novel candidate gene implicated in melanomagenesis.

### iNOS

**Figure 3:**
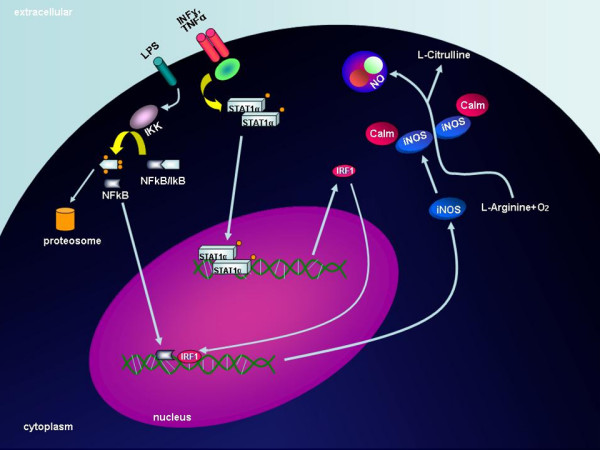
**iNOS pathway**. The functional correlation between the IRF1-activating events (mainly, through an induction regulated by NF-kB, TNF-α, and INF-γ mediators) and expression levels of iNOS is shown. CALM, calmodulin; IkB, inhibitor of kB protein; IKK, inhibitor-of-kB-protein kinase; IRF1, interferon regulatory factor-1; LPS, lipopolysaccharide; NO, nitric oxide; STAT1, signal transducer and activator of transcription 1.

Human melanoma tumors cells are known to express the inducible nitric oxide synthase (iNOS) enzyme, which is responsible for cytokine induced nitric oxide (NO) production during immune responses (Figure 3). The constitutive expression of iNOS in many cancer cells along with its strong association with poor patient survival seems to indicate that iNOS is a molecular marker of poor prognosis or a putative target for therapy [190]. Nitric oxide is a free radical that is largely synthesized by the NO synthase (NOS) enzyme, which exists in three established isoforms: endothelial NOS (eNOS, NOS III) and neuronal NOS (nNOS, NOS I), which are both constitutively expressed and inducible NOS (iNOS, NOS II) which is regulated at the transcriptional level by a variety of mediators (such as interferon regulatory factor-1 [191,192], NF-kB [193,194], TNF-α and INF-γ [195,196] and has been found to be frequently expressed in melanoma [197-200]. The iNOS gene is located at chromosome 17q11.2 and encodes a 131 kDa protein.

In normal melanocytes, the pigment molecule eumelanin provides a redox function supporting an antioxidant intracellular environment. In melanoma cells, a pro-oxidant status has been however reported [195]. Both reactive oxygen species (ROS) and reactive nitrogen oxidants (RNS) can be identified in melanoma. It has been hypothesized that NO may have a different effect on tumors on the basis of its intracellular concentrations. High concentrations of NO might mediate apoptosis and inhibition of growth in cancer cells; conversely, low concentrations of NO may promote tumor growth and angiogenesis [196]. Although the exact function of iNOS in tumorigenesis remains unclear, the overproduction of NO may affect the development or progression of melanoma. It has been shown that the transfection of iNOS gene into murine melanoma cells induces apoptosis, suppresses tumorigenicity, and abrogates metastasis [201,202]. More generally, NO induces apoptosis by altering the expression and function of multiple apoptosis-related proteins (i.e. downregulation of Bcl-2, accumulation of p53, cleavage of PARP [203-209]). The role of iNOS in melanoma progression remains controversial. Higher levels of iNOS have been found in subcutaneous and lymph node metastases of nonprogressive melanoma as compared to metastases of progressive melanoma [210], however, iNOS was found to be expressed to a lesser extent in metastases as compared with nevi and primary melanomas [211]. Nevertheless, the expression of iNOS in lymph nodes and in-transit metastases has been proposed as an indicator of poor prognosis [212].

Finally, nNOS may also play a role in regulating the NO level in cells of melanocytic lineage. The nNOS protein is expressed in the vast majority of melanocytes and cultured melanoma cells, but not in normal melanocytes. However, approximately 49% of benign nevi, 72% of atypical nevi, and 82% of primary malignant melanomas have been reported to express nNOS [213]. The lack of expression of nNOS in normal melanocytes suggests that *de novo *enhanced expression of nNOS may be a marker for an early stage of pigment cell tumor formation, since this variation may lead to an increased level of NO that causes tissue resistance to apoptosis [214].

## Conclusion

Considering the complexity of the above described pathways, probably no individual genetic or molecular alteration is per se crucial; rather the interaction of some or most of such changes are involved in the generation of a specific set of biological outcomes. For melanomagenesis, it is possible to infer that the following alterations are needed:

1. induction of clonal expansion, which is paramount to the generation of a limited cell population for further clonal selection (mutational activation of BRAF or NRAS or amplification of CCND1 or CDK4 may provide this initiating step);

2. modifications to overcome mechanisms controlling the melanocyte senescence, which otherwise would halt the lesion as a benign mole. In melanoma cells both in vitro and in vivo, a change seems to be dramatically required: inactivation of the p16^CDKN2A^-RB pathway (as discussed above, at least 80-90% of uncultured melanomas do show primary inactivation of such a pathway);

3. suppression of the apoptosis. Many of the previously described primary changes suppress the machinery regulating apoptosis allowing for the progression to the vertical growth phase stage (i.e., expression of the AKT antiapoptotic protein was reported to induce the conversion of the radial growth in vertical growth in melanoma).

Despite our attempt to organize the various key molecular alterations involved in melanomagenesis, there may be a relatively large number of alternative primary events, each relatively uncommon on its own, that result in a common secondary outcome, such as upregulation of NFκB and/or variation of the MITF expression levels. The awareness of the existence of such an intracellular web of molecular changes raises a critical question: can some primary alteration in melanoma become suitable as target for therapeutic approaches?

This scenario is further complicated by the fact that the majority of melanomas do not seem to evolve from nevi and only about half of them are associated with dysplastic nevi [215], strongly suggesting that melanoma may mostly arise from normal-appearing skin without following the classical sequential accumulation of molecular events during tumorigenesis. Recently, it has been suggested that melanomas may be derived from transformed melanocyte stem cells, melanocyte progenitors, or de-differentiated mature melanocytes [216,217]. Although the origin of intradermic stem-cells has yet to be determined, it has been postulated that the interaction with the tumor microenvironment (including surrounding and/or recruited fibroblasts and endothelial and inflammatory cells) may induce such cells to transform directly into the various cell variants (normal melanocytes, benign or intermediate proliferating melanocytic cells, malign or metastatic melanoma cells), without progressing through intermediates [217]. In the very near future, the biologic and molecular characterization of melanoma stem cells will also clarify as to whether the well-known drug resistance of melanoma resides in the existence of quiescent or drug-resistant cancer stem cells as well as whether the inhibition of self-renewing cancer stem cells prevents melanoma regrowth.

What we can surely affirm is that targeting a single component in such complex signaling pathways is unlikely to yield a significant anti-tumor response in melanoma patients. For this reason, further evaluation of all known molecular targets along with the molecular classification of primary melanomas could become very helpful in predicting the subsets of patients who would be expected to be more or less likely to respond to specific therapeutic interventions. Now is the time for successfully translating all such research knowledge into clinical practice.

## Competing interests

PAA participated to advisory board from Bristol Myers Squibb and receives honoraria from Schering Plough and Genta.

## Authors' contributions

GP and PAA both conceived of the manuscript, and participated in its design and coordination. All authors either made intellectual contributions and participated in the acquisition, analysis and interpretation of literature data either have been involved in drafting the manuscript and approved the final manuscript.
